# Cortical grey matter volume reduction in people with schizophrenia is associated with neuro-inflammation

**DOI:** 10.1038/tp.2016.238

**Published:** 2016-12-13

**Authors:** Y Zhang, V S Catts, D Sheedy, T McCrossin, J J Kril, C Shannon Weickert

**Affiliations:** 1 Schizophrenia Research Institute, Randwick, NSW, Australia; 2 Schizophrenia Research Laboratory, Neuroscience Research Australia, Randwick, NSW, Australia; 3 School of Psychiatry, University of New South Wales, Sydney, NSW, Australia; 4 Discipline of Pathology, Sydney Medical School, The University of Sydney, Sydney, NSW, Australia

## Abstract

Cortical grey matter volume deficits and neuro-inflammation exist in patients with schizophrenia, although it is not clear whether elevated cytokines contribute to the cortical volume reduction. We quantified cortical and regional brain volumes in fixed postmortem brains from people with schizophrenia and matched controls using stereology. Interleukin (IL)-6, IL-1β, IL-8 and SERPINA3 messenger RNAs (mRNAs) were quantified in the contralateral fresh frozen orbitofrontal cortex. We found a small, but significant reduction in cortical grey matter (1.3% F(1,85)=4.478, *P*=0.037) and superior frontal gyrus (6.5% F(1,80)=5.700, *P*=0.019) volumes in individuals with schizophrenia compared with controls. Significantly reduced cortical grey matter (9.2% F(1,24)=8.272, *P*=0.008) and superior frontal gyrus (13.9% F(1,20)=5.374, *P*=0.031) volumes were found in cases with schizophrenia and ‘high inflammation' status relative to schizophrenia cases with ‘low inflammation' status in the prefrontal cortex. The expression of inflammatory mRNAs in the orbitofrontal cortex was significantly correlated with those in dorsolateral prefrontal cortex (all *r*>0.417, all *P*<0.022), except for IL-8. Moreover, average daily and lifetime antipsychotic intake negatively correlated with cortical grey matter and superior frontal gyrus volumes (all *r*<−0.362, all *P*<0.05). The results suggest that the reduction in cortical grey matter volume in people with schizophrenia is exaggerated in those who have high expression of inflammatory cytokines. Further, antipsychotic medication intake does not appear to ameliorate the reduction in brain volume.

## Introduction

Although neuroimaging studies indicate that many individuals with schizophrenia have reduced cortical brain volumes,^1, 2, 3^ it is debated whether this is due to a pathological process^4^ or medication effects.^5^ In our recent clinical magnetic resonance imaging (MRI) study, we suggested that increased expression of peripheral blood cytokines is associated with Broca's area volume reduction,^6^ leading us to speculate that neuro-inflammation within the brain tissue may contribute to cortical volume reduction.

Previous MRI studies have revealed widespread, and putatively progressive, cortical thinning in some people with schizophrenia, which appears to be related to symptoms and behavioural abnormalities, suggesting that this reduction of cortical tissue is functionally significant.^2^ A recent meta-analysis showed that medicated schizophrenia patients (*n*=3547) had 2.6% smaller total brain volume than controls (*n*=3894).^1^ However, no direct volume measurements of postmortem brain tissue from people with schizophrenia were included, perhaps because only a small number of such studies exist.^7, 8, 9, 10, 11^ Although the previous studies suggest that volume reduction is evident in brains from people with schizophrenia after death, it is not clear to what extent it relates to neuro-inflammation.

Our laboratory has recently identified a link between reduced cortical volume, measured using MRI, and increased levels of inflammatory mediators, especially interleukin (IL)-1β, and vascular endothelial growth factor, in peripheral blood from individuals with schizophrenia.^6, 12^ Higher concentration of pro-inflammatory cytokines (such as IL-6, IL-8 and IL-1β) is a consistent finding in blood,^13^ and in the prefrontal cortex of subsets of people with schizophrenia.^14, 15, 16^ We have linked neuro-inflammation to more severe molecular and cellular neuropathology in schizophrenia, involving exaggerated reduction in interneuron markers and astrogliosis, in our postmortem studies.^14, 17, 18^ This led us to ask whether elevated inflammatory mediators within brain may also be associated with reduced cortical volume.

As it is also possible that antipsychotic treatment is associated with brain volume changes, we considered the potential effect of medications in people with schizophrenia. MRI studies of medicated individuals with schizophrenia demonstrate that antipsychotics may reduce cortical thickness^19^ and volumes.^20, 21^ Rodent studies suggest antipsychotic treatment can induce oxidative stress^22^ and reduce expression of neurotrophins^23^ in brain, which may lead to cell injury or neuronal death and thereby affect brain volume. Thus, antipsychotics intake may negatively affect cortical volume.

The aim of this study was to quantify cortical grey matter, white matter and regional prefrontal cortical volumes in a collection of postmortem brains from patients with schizophrenia and matched controls. We focused on the prefrontal cortex (PFC), because we and others have documented evidence of neuro-inflammation in this region,^14, 15, 16^ and we were seeking to determine whether inflammatory status relates to brain volume in the regions proximal to where neuro-inflammation was identified in tissue. We measured the inflammatory messenger RNAs (mRNAs) in the orbitofrontal cortex (OFC) to determine whether the previously identified dorsolateral prefrontal cortex (DLPFC)-elevated cytokine mRNAs^14^ extend to other prefrontal regions. Moreover, we determined correlations between brain volume and recorded antipsychotic treatment. We hypothesized that schizophrenia patients relative to matched controls would have reduced cortical grey matter volume, particularly affecting prefrontal regions. Furthermore, we hypothesized that people with schizophrenia in a ‘high inflammation' subgroup^14^ have reduced brain volume compared with individuals with schizophrenia without evidence of brain inflammation.

## Materials and methods

### Human postmortem brain section images

New South Wales Brain Tissue Resource Centre (NSWBTRC) in Sydney, Australia, provided photographs of fixed human brain sections and associated data for this study,^24^ which was approved by the Human Research Ethics Committee at the University of New South Wales (HREC No. 12435). Before the brains were fixed, each whole brain was weighed; and the volume measured using fluid displacement^25^ (volumes from three cases are unavailable). One hemisphere of each case was randomly selected to be fixed for 14 days in 15% buffered formalin, previously reported to result in minimal tissue shrinkage (mean: 0.7±0.7%).^26^ Fixed tissue was then sliced at 3 mm in the coronal plane and photographed with a scale bar. Images from 47 people with schizophrenia and 45 control individuals were selected for analysis, with groups matched on demographic variables (Table 1). This sample size was predicted to provide 0.8 power to detect a medium effect size (*d*=0.5) based on variance estimates from a previous publication.^9^ The brains collected by the NSWBTRC undergo a standardized neuropathological examination including screening for neurodegenerative diseases, cerebrovascular disease and microscopic evidence of cerebral hypoxia.^27^ Cases with infectious diseases or poor agonal status (slow death >24 h, for example where death was caused by cancer or chronic pulmonary disease) were excluded.^24, 27, 28^

### Regions of interest

**Figure 1:**
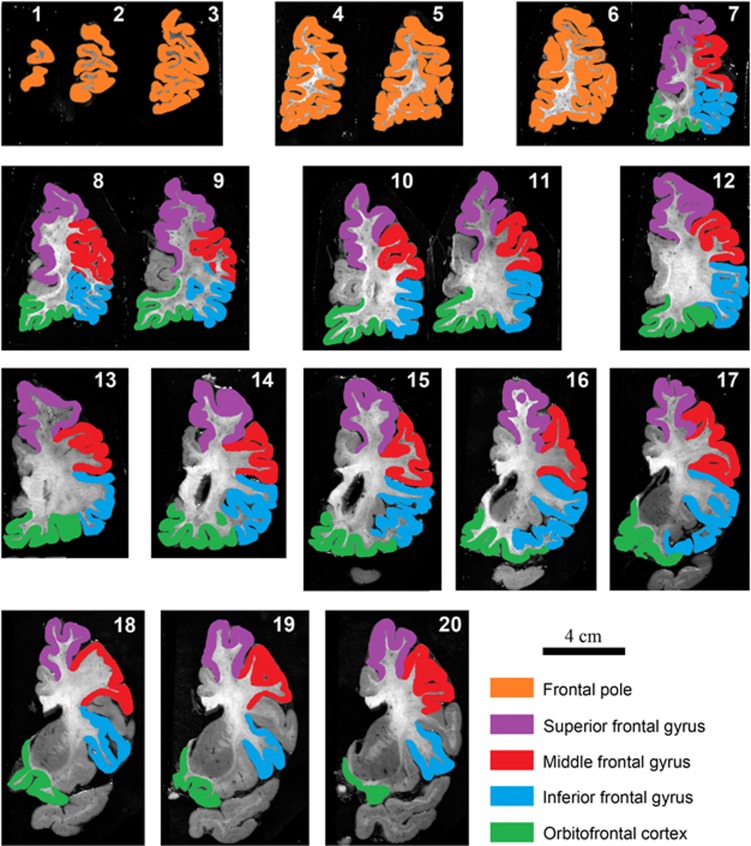
Photographs of 3 mm coronal sections through the prefrontal cortex (PFC) of a formalin-fixed left human brain hemisphere. Images 1 through 20 move progressively in an anterior to posterior direction. PFC starts from the first rostral coronal section, and the last included coronal section for the PFC measurement was the one immediately before the anterior temporal lobe joins with the anterior insula area. Anterior cingulate cortex, insula, precentral gyrus and temporal lobe were not included in the calculations of PFC volume. Frontal pole is from the first anterior section to the section before the appearance of the anterior cingulate (images 1–6). The boundaries between each region of interest were defined by the relevant sulci: superior frontal sulcus (between superior frontal gyrus (SFG) and middle frontal gyrus (MFG)); inferior frontal sulcus (between MFG and inferior frontal gyrus (IFG)); circular insular sulcus (between IFG and insula; and between insula and orbitofrontal cortex (OFC)); cingulate sulcus (between OFC and cingulate; and between cingulate and SFG).

The targets of volume measurement included cortical grey matter, white matter and subregions of the PFC. The cortical grey matter was defined as cerebral cortex including the hippocampus. Total white matter consisted of all white matter including the internal capsule in the striatum. The coronal sections containing PFC were divided into five cortical regions: frontal pole, superior frontal gyrus (SFG), middle frontal gyrus (MFG), inferior frontal gyrus (IFG) and OFC. The boundaries between each region of interest (ROI) were defined by the relevant sulci (Figure 1). Generally, the definitions of PFC and each subregion were based on previous published boundaries,^29, 30, 31^ except for the frontal pole, which spanned from the first anterior section to the section before the appearance of the anterior cingulate (Figure 1). In previous publications, the definition of frontal pole extended to the section before the appearance of the corpus callosum.^29, 30^

### Estimation of brain volume from images using Cavalieri's probe

Total cortical grey and white matter areas were measured on every fifth section (interval 5) starting with a randomly selected section within the first five sections. For other regions of interest, the area was measured on every section where they were evident (interval 1). A transparency with appropriate density of cross points (36.69 mm^2^ per cross) was randomly overlaid on the photographic images. The number of cross points in the target region was counted and recorded. A count was made if the point of intersection at the right angle in the top right quadrant of the cross was placed within the area under investigation. The counted number of cross points was multiplied by the grid size (36.69 mm^2^), the section thickness (3 mm) and the interval between sections (1 or 5). This was divided by 1000 to give the final volume of the ROI in cubic centimetres (cm^3^).^30, 31^

Using one test case supplied by NSWBTRC, YZ and colleagues have measured the cortical grey matter volume and total hemisphere volume six times; then calculated the coefficient of error (CE) (coefficient of error=average standard deviation/average mean). Using this protocol, the coefficient of measurement error was estimated to be 0.019 for total hemisphere volume and 0.018 for cortical grey matter volume. As these coefficients of errors are both less than 0.05, our measurements are considered adequate and reliable.^32^ The brain volume measurements were performed by one investigator (YZ) who was blind to diagnostic categories and confirmed independently on 30% of cases by a second investigator (VSC) who was also blind to diagnostic status of the cases. Intraclass correlation coefficient of average measures between YZ and VSC was 0.982.

### Quantitative PCR of OFC

The mRNA expression of inflammatory modulators was measured in the OFC tissue from 38 schizophrenia and 38 control cases, which has previously been described.^33^ The OFC brain tissue collection included 36 individuals with schizophrenia and 35 controls, also included in our molecular studies of the DLPFC.^14, 34^ RNA isolation, preparation of complementary DNA (cDNA) and quantitative PCR experiments were performed as described previously.^34, 35^ All RNA integrity number (RIN) values were greater than 6 (RIN mean is 7.59±0.83 in controls and 7.51±0.84 in schizophrenia cases).^35^ The transcript levels for inflammatory mRNAs (SERPINA3, IL-6, IL-8 and IL-1β) were measured by quantitative real-time PCR in each OFC sample using an ABI Prism 7900HT Fast Real Time PCR system with a 384-well format (Applied Biosystems, Foster City, CA, USA) and TaqMan Gene Expression Assays (Applied Biosystems). The samples were run with a seven-point standard curve using serial dilutions of pooled cDNA derived from a representative sample of subjects (three controls and three patients). The no-template control did not produce a signal in any assay. All the amplifications from each subject were performed in triplicate and relative quantities were determined from the standard curve. The mRNA expression levels of inflammatory genes were measured using TaqMan probe: SERPINA3 (Hs003153674_m1), IL-6 (Hs00174131_m1), IL-8 (HS00174103_m1), and IL-1β (Hs01555410_m1); with ACTB (Hs99999903_m1), GAPDH (Hs99999905_m1), TBP (Hs00427621_m1) and UBC (Hs00824723_m1) used as normalizing housekeeping genes. The cycling conditions and reaction amounts were the same as for the previous DLPFC cytokine study.^14^

### Statistical analysis

The data analysis was performed using IBM SPSS Statistics 22 (Sydney, NSW, Australia). Normal distributions of data were tested by the Kolmogorov–Smirnov and the Shapiro–Wilk tests. The comparison analyses for the volume of each ROI between any groups (diagnosis; low or high level inflammation;^14^ ‘yes' or ‘no' history of illicit drugs and smoking) were performed by analysis of covariance. We used total hemisphere volume as a co-variate to correct for gender differences in brain volume,^36^ thereby increasing our statistical power; and age was included as a co-variate, because it is known to affect brain volume.^37^ Before analysis of covariance, as total hemisphere volume was chosen as co-variate, the ROI volume was graphed against total hemisphere volume;^36^ and a 95% confidence interval boundary imposed for each diagnostic group separately to identify measurement outliers (average *n*=2 outliers per region per diagnostic group; Supplementary Figure 1).

The DLPFC in the contralateral frozen hemisphere of a subset of the brains in which volume were determined have been investigated previously using molecular techniques (24 controls, 28 people with schizophrenia, Supplementary Table 1).^34^ This includes measurement of inflammatory mediator mRNA expression, which were used to identify those brains with ‘high or low' inflammatory levels through two-step recursive clustering analysis.^14^ In our previous paper, we performed next-generation sequencing on the DLPFC transcriptome (33 105 transcripts) mapped from 20 individuals with schizophrenia and 20 controls. Ingenuity analysis identified five networks and five biological functions that were dysregulated in schizophrenia, in which a neuro-inflammatory pathway reoccurred. The seven most-changed inflammatory genes were followed up by quantitative PCR in an expanded cohort of 74 individuals in total. Using cluster analysis, we found that in the DLPFC, four of the seven inflammatory markers (SERPINA3, IL-6, IL-1β and IL-8 mRNA levels) significantly predicted inflammatory cluster membership. The overall model quality for the DLPFC cluster analysis was required to be >0.5, with predictors removed if they did not contribute >0.5 to the model on a scale of 0 to 1.0. In the current OFC study, we therefore measured and analysed SERPINA3, IL-6, IL-1β and IL-8 mRNA levels using quantitative PCR. The initial non-normally distributed data were log10 (SERPINA3, IL-8, and IL-1β) or inverse (IL-6) transformed. Similar to the DLPFC clustering, the inflammatory subgroups based on OFC inflammatory mediators' measurements were determined using a recursive two-step cluster analysis using all the available values. The characteristics of this subgroup were identified by using *t*-tests for normally distributed continuous variables.

Outliers in diagnostic groups were defined by Grubbs test after data normalization and excluded from further analysis. We used analysis of variance to investigate differences between OFC inflammatory cytokines in the different inflammatory subgroups. The five control individuals with high inflammation were excluded from statistical analysis owing to the small group size. All the tests were two-tailed.

## Results

### Stereological measures of brain volumes correlated with other indices of hemisphere volume

In the whole cohort, the brain weight correlated positively with all brain volumes measured (all 0.374<*r*<0.675, *P*<0.001). The total brain displacement volume also significantly correlated with brain weight (*r*=0.978, *P*<0.001) and all measured ROI volumes (all 0.376<*r*<0.723, *P*<0.001; Supplementary Table 2).

### The effect of demographic and perimortem factors on brain volume measures

In the whole cohort, age correlated negatively with the brain weight (*r*=−0.391, *P*<0.001), displacement volume (*r*=−0.355, *P*=0.001), and all the brain volumes measured in this study (all *r*<−0.214, *P*<0.044). The tissue pH correlated positively with the displacement volume (*r*=0.220, *P*=0.039; Supplementary Table 2). There was no statistically significant difference for the measured cortical volumes between the agonal state groups^27^ (all *P*>0.15).

In the whole cohort (Supplementary Table 3), as expected, males had larger brain weights (F(1,89)=48.703, *P*<0.001), displacement volumes (F(1,86)=36.118, *P*<0.001) and total hemisphere volumes (F(1,89)=9.540, *P*=0.003) than females (co-varied with age). When co-varied with age and total hemisphere volume, all the measured cortical and regional volumes did not show any differences between left and right hemispheres (Supplementary Table 3).

### Brain volumes in people with schizophrenia

**Figure 2:**
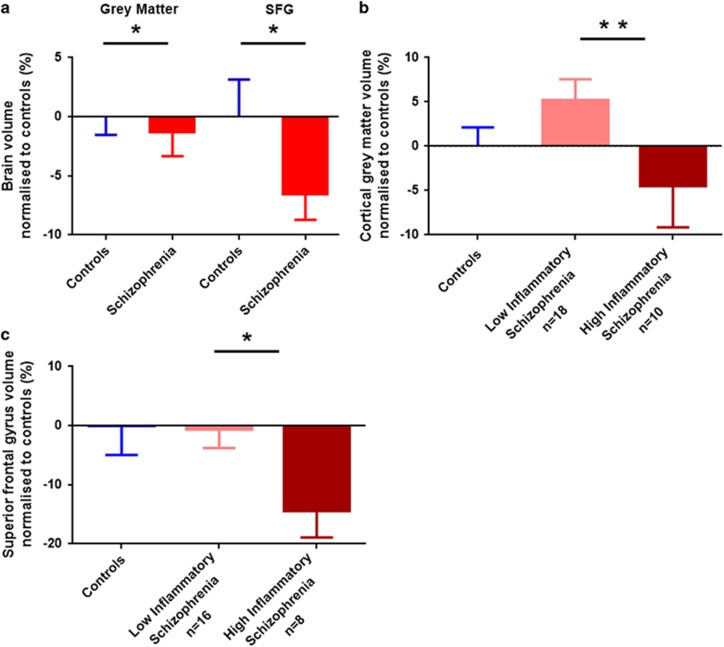
Analyses of cortical grey matter and superior frontal gyrus (SFG) volumes in postmortem brains from control individuals (blue) and people with schizophrenia (red) (**a**). Cortical grey matter (**b**) and SFG (**c**) volumes in individuals with schizophrenia who had increased expression of inflammatory markers (dark red) were significantly reduced compared with ‘low inflammation' schizophrenia (pink). All volume data were normalized to the mean of control volume; error bars denote standard error of the mean (*P<0.05, **P<0.01).

The gross measures of brain volume (brain weight, displacement volume and total hemisphere volume) did not differ between individuals with schizophrenia and the controls (Supplementary Table 3), likely owing to the large population variance in these measures. However, compared with unaffected controls, the total cortical grey matter volume was significantly reduced by 1.3% in people with schizophrenia (F(1,85)=4.478, *P*=0.037; Figure 2a). The biological variance (standard deviation divided by the group mean) in total cortical grey matter was 14% for schizophrenia and 10% for control cases, which is considerably greater than the measured error of ~2%. No significant differences between individuals with schizophrenia and controls were found for subcortical grey matter (F(1,85)=0.097, *P*=0.756) or white matter (F(1,85)=3.364, *P*=0.070) volumes (Supplementary Table 3).

Overall, there was no significant difference in the PFC volumes between controls and people with schizophrenia (F(1,79)=0.144, *P*=0.706). For the subregions of the PFC, a significant difference in SFG was observed, where people with schizophrenia had a 6.5% smaller volume relative to controls (F(1,80)=5.700, *P*=0.019; Figure 2a). The volumes of the frontal pole, MFG, IFG and OFC did not differ significantly between controls and people with schizophrenia (Supplementary Table 3).

### Grey matter and SFG volume changes were related to inflammation

Using the inflammatory cluster defined for DLFPC tissue in our previous publication,^14^ we found significant effects of inflammatory status on both total cortical grey matter and SFG volumes. People with schizophrenia and ‘high inflammation' (elevated cytokine mRNAs) had significantly smaller (9.2%) cortical grey matter than those with ‘low inflammation' (F(1,24)=8.272, *P*=0.008; Figure 2b). There was a significantly smaller (13.9%) SFG volume in the ‘high inflammation' schizophrenia group than the ‘low inflammation' schizophrenia group (F(1,20)=5.374, *P*=0.031; Figure 2c).

The volumes of cortical grey matter and SFG were significantly negatively correlated with IL-6 and SERPINA3 mRNAs for both the whole cohort and the schizophrenia group only (all −0.700<*r*<−0.341, *P*<0.01; Supplementary Table 4 and Supplementary Figure 2). We also found that SFG volume was significantly negatively correlated with IL-1β mRNA levels in the schizophrenia group only (*r*=−0.563, *P*<0.01). However, there were no significant correlations between the levels of IL-8 mRNA and cortical grey matter or SFG volumes (both *r*<0.149, *P*>0.05).

### Inflammatory mediator mRNA expressions in OFC

In the whole OFC cohort, SERPINA3 and IL-6 mRNAs were significantly negatively correlated with age and pH (all −0.553<*ρ*<−0.141, 0.001<*P*<0.032) and IL-6 mRNA was also significantly correlated with RIN (*ρ*=−0.264, *P*=0.022). Furthermore, in the schizophrenia group, SERPINA3 (*ρ*=0.397, *P*=0.016) and IL-6 (*ρ*=0.464, *P*=0.004) mRNAs were significantly positively correlated with antipsychotic (lifetime) intake. Moreover, IL-6 mRNA was also significantly correlated with antipsychotic (daily mean) intake (*ρ*=0.399, *P*=0.016). All the other correlations between OFC inflammatory markers and demographics were not statistically significant (all −0.205<*ρ*<0.311, 0.065<*P*<0.962; Supplementary Table 5).

For individuals with schizophrenia, each inflammatory mRNA in OFC tissue was significantly correlated with its expression in the DLPFC tissue (SERPINA3 *r*=0.723, *P*<0.001; IL-1β *r*=0.744, *P*<0.001; IL-6 *r*=0.686, *P*<0.001; IL-8 *r*=0.417, *P*=0.022).

**Figure 3:**
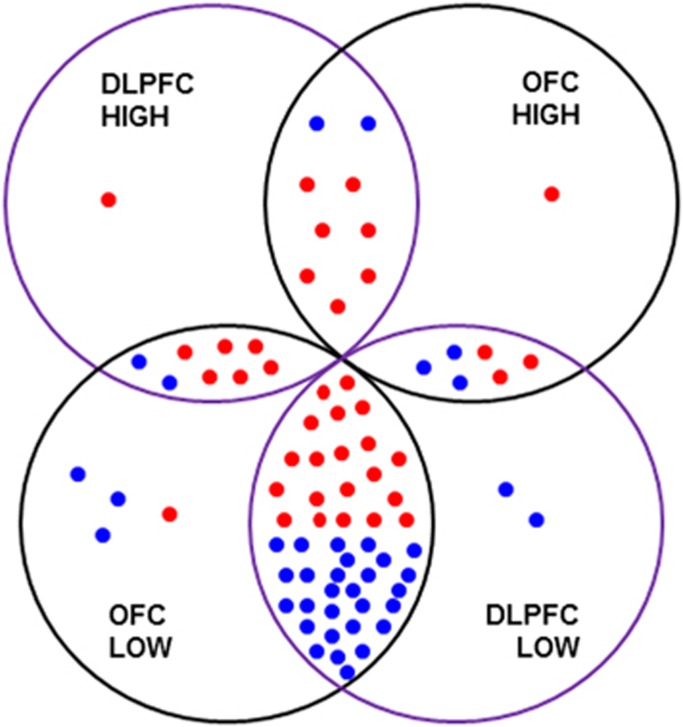
The overlap between ‘high or low inflammation' cases in the orbitofrontal cortex (OFC) and dorsolateral prefrontal cortex (DLPFC) cohorts. There are 36 schizophrenia and 35 control cases in common between the OFC and DLPFC cohort (in the overlapping areas between the four circles). Five control and three schizophrenia cases are unique to either the DLPFC or OFC cohorts. Their inflammatory statuses are indicated in the four circles (‘HIGH': high inflammation; ‘LOW': low inflammation). Seven schizophrenia and two control cases were identified as ‘high inflammation' in both the DLPFC and OFC cohort. Twenty schizophrenia and 28 control cases were identified as ‘low inflammation' in both the DLPFC and OFC cohort. However, six schizophrenia and two control cases were identified as ‘high inflammation' in the DLPFC but as ‘low inflammation' in the OFC cohort. Three schizophrenia and three control cases were identified as ‘high inflammation' in the OFC but ‘low inflammation' in the DLPFC cohort. ‘Blue dot: control case; red dot: schizophrenia case.'

Using two-step cluster analysis, we found that 16 out of 76 individuals in the OFC cohort were defined by high IL-1β and SERPINA3 mRNA expression in the OFC (IL-6 and IL-8 were removed because they did not contribute >0.5 to the model). In the OFC cohort, 29% of schizophrenia cases were determined to have high levels of inflammatory cytokine expression; whereas 13% of controls had ‘high inflammation'. There was considerable overlap in the identified cases with high and low levels of inflammation between the DLPFC and OFC cohorts (Figure 3).

**Figure 4:**
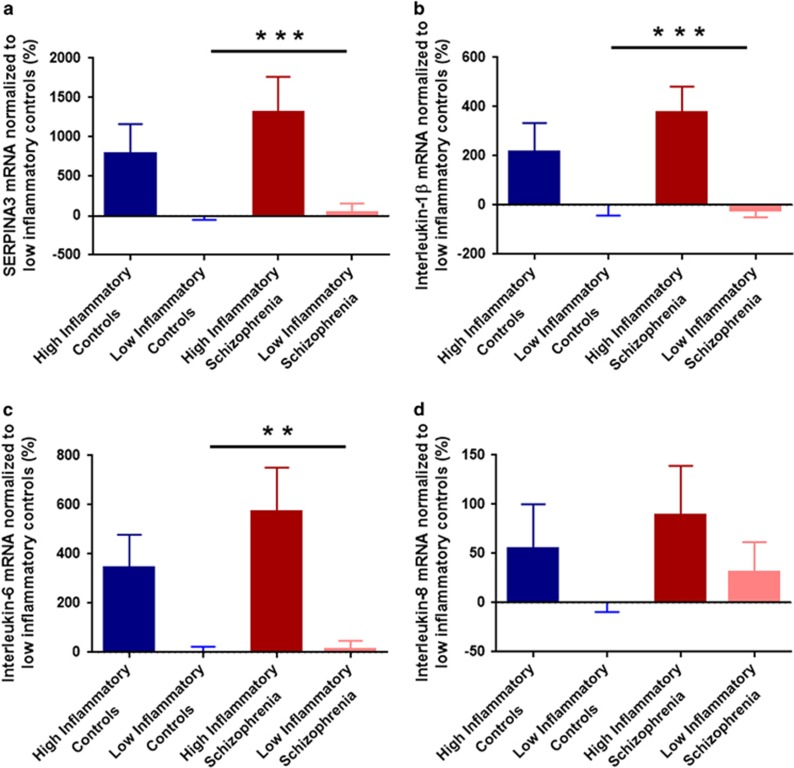
Inflammatory messenger RNA (mRNA) expressions in the orbitofrontal cortex (OFC) measured by quantitative PCR. All expression data were normalized to low-inflammation controls (%). Data from the five control individuals with high inflammation are shown here for illustrative purposes but were excluded from statistical analysis owing to the small group size. (**a**) SERPINA3 mRNA; (**b**) IL-1β mRNA; (**c**) IL-6 mRNA; (**d**) IL-8 mRNA. Blue bars indicate controls and red bars are individuals with schizophrenia, error bars denote standard error of the mean (**P<0.01, ***P<0.001). IL, interleukin.

Inflammatory mediator mRNAs in OFC differed significantly between low-inflammatory controls and low- and high-inflammatory schizophrenia (except IL-8; (SERPINA3 F(2,68)=41.036, *P*<0.001; IL-1β F(2,68)=45.972, *P*<0.001; IL-6F(2,67)=8.319, *P*=0.001; IL-8F(2,68)=2.976, *P*=0.058); Figure 4 and Supplementary Figure 3).

### Cortical grey matter and SFG volumes changes were associated with antipsychotics intake, but not with illicit drug use or smoking

Volumes of cortical grey matter and SFG were significantly negatively correlated with ‘chlorpromazine equivalents lifetime' and ‘chlorpromazine equivalents daily mean' doses (all *r*<−0.362, *P*<0.05; Supplementary Table 4). When partial correlations, controlling for daily mean chlorpromazine equivalent dose, were applied to test the robustness of the relationship between cytokines and prefrontal cortex volume in people with schizophrenia, the correlation between SFG volume and IL-6 mRNA levels reduced from −0.700 (*P*<0.001) to −0.441 (*P*=0.024), but remained statistically significant. This suggests that even when statistically controlling for the medication dose, IL-6 expression accounts for 19.4% of the variance in grey matter volume in people with schizophrenia.

By the analysis of covariance analysis (co-varying with age and total hemisphere volume), there was no significant difference of cortical grey matter volumes (F(1,21)=0.277, *P*=0.604) and SFG (F(1,19)=0.109, *P*=0.745) between people with schizophrenia who had and who did not have a history of illicit drug use. Similarly, smoking history did not appear to affect the cortical grey matter (F(1,38)=0.011, *P*=0.916) and SFG (F(1,35)=0.058, *P*=0.811) volumes in people with schizophrenia in our study.

## Discussion

In this study, we found that cortical grey matter and SFG volumes were significantly smaller in schizophrenia subjects than controls, and that neuro-inflammatory status has a significant association with cortical grey matter reduction in schizophrenia. Moreover, this study suggests that smaller cortical volumes in schizophrenia are associated with greater intake of antipsychotics. Thus, this study supports the suggestion that cortical grey matter changes may have both an inflammatory basis and a relationship to antipsychotic exposure, and does not support that antipsychotics can ameliorate brain volume reduction in schizophrenia.

The postmortem human brain volume measures described here concur with expectations. Previously published measurements in controls of similar age to the individuals in the present study show that the cortical grey matter volume is 54% of total hemisphere volume,^29, 30, 31^ similar to our result of 56% cortical grey matter to total hemisphere volume. Also, PFC volume of controls is about 72.2 ml,^30^ which is close to our result of approximately 75 ml. These similar results support the reliability of our measurements. However, our study and the previous studies,^30, 31^ differ in the definition of the frontal pole, resulting in our measured frontal pole volume (3% of total hemisphere volume) being smaller than previous reports (7.3% of total hemisphere volume).^31^ According to anthropology and brain anatomy, Brodmann area 10 is the major area in the frontal pole of human brain,^38^ accounting for ~1.2% of brain volume,^39^ which is close to the frontal pole percentage estimated in the current study, and suggests that previous definitions of frontal pole may have been overly inclusive.

Only few postmortem volume measurements of brains from people with schizophrenia have been published. A stereology volume study of postmortem brains from 14 individuals with schizophrenia and 19 unaffected controls^9^ found a significant 12% decrease in frontal grey matter volume in schizophrenia subjects compared with control subjects. This is considerably greater than the 1.3% differences in cortical grey matter volume between diagnostic groups in the present study. Our result of a small but significant cortical grey matter reduction in schizophrenia appears more consistent with MRI studies finding 2.9–3.4% reduction in patients relative to healthy controls.^40, 41^ However, our study suggests that depending on the percentage of people who have schizophrenia and high inflammation sampled within a cohort, we may expect quite a wide range in the brain volume loss reported especially for small samples, as we find up to 9.2% loss in the high-inflammation subgroup alone and if this group was oversampled, it would lead to a higher estimate of brain tissue loss on average. Some MRI studies also report that people with schizophrenia have 7–8% smaller SFG volumes,^42, 43^ consistent with our results where a 6.5% smaller volume of this dorsal prefrontal cortex was found in people with schizophrenia. However, those previous MRI studies also found other PFC subregional volumes (MFG and IFG) to be reduced,^42, 43^ which we did not. Indeed MRI^1, 2^ volumetric studies of schizophrenia have observed smaller grey matter volumes in a number of cortical regions, suggesting brain volume changes in schizophrenia are likely to be global and not restricted to just one gyrus.

However, not all brain volume studies of schizophrenia find significant results and the percentage of brain volume reduction are not always equivalent. One possible reason is that brain volume reductions may be exaggerated in certain brain regions or may be clearly present in only some people with schizophrenia due to the heterogeneity of the disease. In support of this, we find exaggerated volume changes in a subset (40%) of people with schizophrenia with high levels of inflammatory mediators (‘high inflammatory' biotype).^6, 14^ Although this is a sizeable minority of cases, differential sampling of this inflammatory subset within other cohorts especially those with a limited number of subjects might explain the variable results.

A major finding of our study is that individuals with ‘high inflammation' schizophrenia had smaller cortical brain volume than individuals with ‘low inflammation' schizophrenia, although the underlying reasons for this association remain unclear. We also found that IL-6, SERPINA3 and IL-1β mRNA levels had significantly negative relationships with the volume reduction in schizophrenia, suggesting that increases in at least these three factors are linked to reduced brain volumes. However, the correlation analysis alone does not separate cause from consequence. In the brain, IL-6 and IL-1β are produced by neurons, astrocytes, microglia and oligodendrocytes.^44, 45, 46, 47^ These same cell types also express the IL-6 and IL-1β receptor and thus are capable of responding to the two cytokines.^44, 45, 48, 49^ Although normal physiological levels of IL-6 and IL-1β appear to be important for processes such as long-term potentiation and neurogenesis, upregulation of these cytokines in inflammatory conditions is accompanied by oxidative stress and neurodegenerative events.^50, 51^ SERPINA3 (also known as alpha-1-antichymotrypsin) is a serpin peptidase inhibitor.^52^ In brain, SERPINA3 expression and secretion appears to occur mainly in response to pro-inflammatory cytokine signalling in activated astrocytes and microglia.^53, 54, 55, 56^ The increased expression of SERPINA3 is associated with neurodegeneration,^56, 57^ and has consistently been observed in postmortem tissue from individuals with schizophrenia by us ^14, 15^ and others.^58, 59^ Furthermore, even though this study did not identify a significant correlation between IL-8 and brain volume, IL-8 may still be a potential factor contributing to brain damage. In the brain, IL-8 (also known as CXCL8) is a chemoattractant produced by microglial cells.^60^ Elevated IL-8 is associated with abnormal neurodevelopment,^61^ which implicates IL-8 in perinatal brain damage;^62^ the mechanism of which is not known. Taken together, the present results suggest that further study of the cellular source of the increased pro-inflammatory mediators and potential consequence of these increases on neuronal and glial health and the integrity of cortical volume in the context of schizophrenia risk factors is warranted.

The previously defined ‘high inflammation' schizophrenia group was based on a single brain region, the DLPFC.^14^ It was unclear whether expression of inflammatory mediators in this one cortical area reflects the inflammatory levels in the other regions or even the whole brain. For practical reasons, we cannot investigate the inflammation changes across the whole brain simultaneously as may be more achievable with imaging techniques. In fact, by using activated microglia as neuro-inflammatory markers, neuro-inflammation has previously been identified across multiple brain regions in individuals with schizophrenia using positron emission tomography (PET) imaging,^63, 64^ though not every study finds an increase.^65^ Using the first-generation translocator protein 18 kDa (TSPO) ligand [^11^C]PK11195, Doorduin *et al.*^64^ found numerically increased binding potentials in the frontal, occipital, temporal and parietal lobes, although this only reached statistical significant in the temporal lobe. However, from the data presented it is not clear whether the same individuals have increased binding potential across the cortex or whether different individuals contribute to the increase in different regions. Using the second-generation TSPO PET ligand [^11^C]PBR28, Bloomfield *et al.*^63^ found elevated binding across the frontal and temporal lobe, and the total grey matter. Inspection of representative images in the Bloomfield *et al.* publication suggests elevated PBR28 binding across the cortex, as opposed to discrete cortical regions, in individuals with schizophrenia, suggesting the inflammation is global. Given that we have previously identified striking pathology in the OFC of individuals with schizophrenia, including increased density of interstitial white matter neurons^33^ and decreased expression of interneuron markers,^18, 33, 66^ we choose to extend the measurement of inflammatory cytokines to the OFC. Similar results to those in the DLPFC were found with increased pro-inflammatory mediators that could be used to identify those in a ‘high inflammatory' state. These data suggest that cytokine changes are present across multiple brain regions in schizophrenia. Thus, our DLPFC and OFC cytokine data may index more widespread cortical inflammation levels.

The current study identified 29% of schizophrenia cases in the ‘high inflammation' cluster based on elevated OFC cytokines, which is different from our previous findings of 38% based on DLPFC;^14^ from 46% based on our second DLPFC study;^15^ and from 40% based on our mRNA analysis of peripheral blood.^6^ Moreover, a recently published human postmortem study identified elevated IL-6, IL-8 and IL-1β mRNA expression levels in schizophrenia PFC tissue from a large cohort of 62 schizophrenia cases and 62 controls, which showed a wide distribution of cytokine levels.^16^ The data presented in this independent study^16^ also suggests that some, but not all individuals with schizophrenia display elevated neuro-inflammation in the PFC; however, a high-inflammatory subset was not determined. These studies suggest that there is a ‘high inflammation' subgroup in people with schizophrenia, and while the percentage of this subgroup is not yet clear the average percentage appears to be 38%. However, cluster analysis is only one way to demonstrate that subgroups exist in a cohort, and clustering is sensitive to which markers are included/excluded but it cannot identify definitive cut-offs for defining individuals to each subgroup. Although our results suggest that at least two subgroups exist, further work in refining the molecular markers used to define this group and their relative importance in determining group membership may be needed to make these markers more generally applicable.

Our previous work in classifying inflammatory subgroups of individuals with schizophrenia appears to be valid based on other biological measures that distinguish individuals in these groups. We found that high expression of inflammatory cytokines in schizophrenia is linked to increased expression of glial fibrillary acidic protein and hypertrophic astrocyte morphology.^17^ Astrogliosis could be a compensatory response to the process causing the brain volume reduction, as it often occurs after neuron damage. This suggests that neurodegenerative events can be identified in the schizophrenia brain and that some mild form of tissue reduction may occur and be exaggerated in, or restricted to, a subset of people with schizophrenia.

In the last decade, antipsychotic treatment has been hypothesized to be a major contributor to brain volume reduction in schizophrenia.^20, 21^ As MRI studies can recruit individuals with first-episode schizophrenia pre- or post-treatment, they can more clearly determine the effect of antipsychotics on brain volumes.^2, 5^ Although the current postmortem study does not include first-episode cases, we still found that grey matter volumes were significantly negatively correlated with antipsychotic intake, suggesting that antipsychotic treatment may contribute to brain volume reduction. Alternatively, it may indicate that brain volume changes are associated with a more severe illness that demands higher dose of antipsychotics treatment.

Our study suggests that both inflammatory mediators and antipsychotic treatment could affect cortical brain volumes in schizophrenia. In this regard, it is interesting to note that *in vitro*^67^ and *in vivo*^68^ studies find that antipsychotic treatment may modulate levels of anti- and pro-inflammatory cytokines. This suggests a model whereby antipsychotic treatment interacts with a pathological process (inflammation), which affects the grey matter volume. Although speculative, this relationship between inflammation and antipsychotics could be further tested in animal studies.

The study is limited in several respects, which should be taken into consideration when interpreting findings. First, since all analysed data came from postmortem brain, some perimortem factors of the study samples could not be controlled. These include the stage of illness, antipsychotic intake and comorbidities such as smoking and metabolic syndrome. As the postmortem cohort includes cases at multiple stages of illness, we cannot determine when the changes (such as oxidative stress, neuro-inflammation, interneurons damage, and so on) occur; and different stages of illness may show different changes. Second, the NSWBTRC has limited information on what type of illicit drug individuals used during their life but indicated which cases have a history of illicit drug use. We note that in the high-inflammation schizophrenia subgroup, four individuals had a history of illicit drugs use, but we do not know which type of drug it was. Although it is possible that some particular type of illicit drugs may have effects on the brain volume and neuro-inflammatory changes, we cannot consider it in our analysis. For instance, cannabis can have anti-inflammatory actions,^69^ so future studies could be better designed to take cannabis exposure into account. Third, when postmortem brain tissues were prepared, one hemisphere (formalin-fixed) was chosen for volume measurement and the other (frozen) was for molecular investigation. The hemispheres were randomised to fixation or freezing to minimize the effect of hemisphere differences, but we cannot exclude an effect of hemispheric differences on our results. A further limitation of our study is a potential differential effect of tissue shrinkage associated with the fixation protocol utilized. It is possible that factors such as medication, inflammation status, smoking or drug usage may alter the effect of formalin on brain tissue. This could not be assessed in the current study as the pre-fixation indices of brain volume (brain weight and displacement volume) included the brain stem and the post-fixation indices of brain volume (by *Cavalieri's* probe on photographs) did not. However, our measures obtained after fixation strongly correlated with pre-fixation measures of displacement volumes, in both the control and schizophrenia groups, indicating that a diagnosis of schizophrenia was not associated with a differential effect of fixation on tissue volume. Because the volume study and the inflammatory studies were based on different but overlapping cohorts, with only about 57% of cases with brain volume measurements and molecular data, our study had relatively low statistical power and meant that the high-inflammation schizophrenia group for whom we had volume measures only included 10 cases and that high-inflammation control group was too small for meaningful statistical analysis. The sample size necessitated only examining a small number (14) of molecular correlations rather than 100 or thousands, which would increase the risk of type II error. Previous studies found that illicit substance abuse and tobacco smoking reduces cortical volumes.^70, 71^ Illicit substance abuse and smoking are very common in patients with schizophrenia,^72^ but we did not find an effect of either on cortical volume in people with schizophrenia in our study, perhaps partly because of low statistical power, but alternatively that the effects of substance abuse and smoking on brain volume are not larger than effects of schizophrenia and/or inflammation status.

In conclusion, we found that smaller cortical volume in individuals with schizophrenia is associated with neuro-inflammation. However, the mechanism underlying volume reduction is still not fully elucidated, and may also involve treatment with antipsychotics. Future studies will seek to relate the volume deficits to other cellular and molecular factors associated with schizophrenia neuropathology,^73^ to further support the link between elevated cytokines and brain volume reduction and to extend knowledge of the putative mechanisms and cells involved. The present study also provides evidence at the level of brain tissue that clinical use of anti-inflammatory medications may preserve tissue volume of prefrontal cortex of people with schizophrenia and suggests that structural MRI studies could be used alongside anti-inflammatory medications to test this possibility.

## Figures and Tables

**Table 1:** Brain volume cohort description

	*Controls*			*Schizophrenia*			*Statistics*			
	N	*Mean*	*s.d.*	N	*Mean*	*s.d.*	X^*2*^	t	*df*	P
Male	31	—	—	31	—	—	0.090	—	1	0.764
Female	14	—	—	16	—	—				
Fixed left hemisphere	27	—	—	24	—	—	0.743	—	1	0.389
Fixed right hemisphere	18	—	—	23	—	—				
Age (years)	—	52.6	13.7	—	51.9	13.5	—	−0.242	90	0.810
PMI (h)	—	28.7	12.2	—	33.4	16.2	—	1.569	90	0.120
Brain weight (g)	—	1431	143	—	1411	152	—	−0.672	90	0.503
Tissue pH	—	6.4	1.0	—	6.5	0.2	—	0.535	89	0.594
DLPFC: high inflammation	2	—	—	10	—	—	5.458	—	1	0.024
DLPFC: low inflammation	22	—	—	18	—	—				
Inflammation unknown^a^	21	—	—	19	—	—	—	—	—	—
Age of onset	—	—	—	—	22.8	7.0	—	—	—	—
Duration of illness (years)	—	—	—	—	29.1	13.7	—	—	—	—
Antipsychotics daily mean (mg)	—	—	—	35	744	502	—	—	—	—
Antipsychotics lifetime (g)	—	—	—	35	8872	7903	—	—	—	—
Smoking	19	—	—	30	—	—	0.089	—	1	0.105
No smoking	17	—	—	12	—	—				
Smoking unknown^a^	9	—	—	5	—	—	—	—	—	—
Illicit drug	0	—	—	11	—	—	0.001	—	1	0.001
No illicit drug	20	—	—	14	—	—				
Illicit drug unknown^a^	25	—	—	22	—	—	—	—	—	—

Abbreviations: DLPFC, dorsolateral prefrontal cortex; PMI, postmortem interval.

a Unknown cases were not entered into statistics.

Antipsychotics data are chlorpromazine equivalents.

## References

[bib1] Haijma SV, Van Haren N, Cahn W, Koolschijn PC, Hulshoff Pol HE, Kahn RS. Brain volumes in schizophrenia: a meta-analysis in over 18 000 subjects. Schizophr Bull 2013; 39: 1129–1138. 23042112 10.1093/schbul/sbs118 PMC3756785

[bib2] Pantelis C, Velakoulis D, McGorry PD, Wood SJ, Suckling J, Phillips LJ et al. Neuroanatomical abnormalities before and after onset of psychosis: a cross-sectional and longitudinal MRI comparison. Lancet 2003; 361: 281–288. 12559861 10.1016/S0140-6736(03)12323-9

[bib3] Honea R, Crow TJ, Passingham D, Mackay CE. Regional deficits in brain volume in schizophrenia: A meta-analysis of voxel-based morphometry studies. Am J Psychiatry 2005; 162: 2233–2245. 16330585 10.1176/appi.ajp.162.12.2233

[bib4] Pantelis C, Yücel M, Wood SJ, Velakoulis D, Sun D, Berger G et al. Structural brain imaging evidence for multiple pathological processes at different stages of brain development in schizophrenia. Schizophr Bull 2005; 31: 672–692. 16020551 10.1093/schbul/sbi034

[bib5] Ho BC, Andreasen NC, Ziebell S, Pierson R, Magnotta V. Long-term antipsychotic treatment and brain volumes: a longitudinal study of first-episode schizophrenia. Arch Gen Psychiatry 2011; 68: 128–137. 21300943 10.1001/archgenpsychiatry.2010.199 PMC3476840

[bib6] Fillman SG, Weickert TW, Lenroot RK, Catts SV, Bruggemann JM, Catts VS et al. Elevated peripheral cytokines characterize a subgroup of people with schizophrenia displaying poor verbal fluency and reduced Broca's area volume. Mol Psychiatry 2016; 21: 1090–1098. 26194183 10.1038/mp.2015.90 PMC4960447

[bib7] Dwork AJ. Postmortem studies of the hippocampal formation in schizophrenia. Schizophr Bull 1997; 23: 385–392. 9327505 10.1093/schbul/23.3.385

[bib8] Highley JR, McDonald B, Walker MA, Esiri MM, Crow TJ. Schizophrenia and temporal lobe asymmetry. A post-mortem stereological study of tissue volume. Brit J Psychiatry 1999; 175: 117–134. 10.1192/bjp.175.2.127 10627794

[bib9] Selemon LD, Kleinman JE, Herman MM, Goldman-Rakic PS. Smaller frontal gray matter volume in postmortem schizophrenic brains. Am J Psychiatry 2002; 159: 1983–1991. 12450946 10.1176/appi.ajp.159.12.1983

[bib10] Bogerts B, Falkai P, Haupts M, Greve B, Ernst S, Tapernon-Franz U et al. Post-mortem volume measurements of limbic system and basal ganglia structures in chronic schizophrenics: initial results from a new brain collection. Schizophr Res 1990; 3: 295–301. 2282334 10.1016/0920-9964(90)90013-w

[bib11] Bogerts B, Meertz E, Schönfeldt-Bausch R. Basal ganglia and limbic system pathology in schizophrenia: a morphometric study of brain volume and shrinkage. Arch Gen Psychiatry 1985; 42: 784–791. 4015323 10.1001/archpsyc.1985.01790310046006

[bib12] Pillai A, Howell KR, Ahmed AO, Weinberg D, Allen KM, Bruggemann J et al. Association of serum VEGF levels with prefrontal cortex volume in schizophrenia. Mol Psychiatry 2016; 21: 686–692. 26169975 10.1038/mp.2015.96

[bib13] Miller BJ, Buckley P, Seabolt W, Mellor A, Kirkpatrick B. Meta-analysis of cytokine alterations in schizophrenia: clinical status and antipsychotic effects. Biol Psychiatry 2011; 70: 663–671. 21641581 10.1016/j.biopsych.2011.04.013 PMC4071300

[bib14] Fillman SG, Cloonan N, Catts VS, Miller LC, Wong J, McCrossin T et al. Increased inflammatory markers identified in the dorsolateral prefrontal cortex of individuals with schizophrenia. Mol Psychiatry 2013; 18: 214–222. 10.1038/mp.2012.110 22869038

[bib15] Fillman SG, Sinclair D, Fung SJ, Webster MJ, Weickert CS. Markers of inflammation and stress distinguish subsets of individuals with schizophrenia and bipolar disorder. Transl Psychiatry 2014; 4: e365. 24569695 10.1038/tp.2014.8 PMC3944638

[bib16] Volk DM, Chitrapu A, Edelson JR, Roman KM, Moroco AE, Lewis DA. Molecular mechanisms and timing of cortical immune activation in schizophrenia. Am J Psychiatry 2015; 172: 1112–1121. 26133963 10.1176/appi.ajp.2015.15010019 PMC5063256

[bib17] Catts VS, Wong J, Fillman SG, Fung SJ, Weickert CS. Increased expression of astrocyte markers in schizophrenia: association with neuroinflammation. Aust N Z J Psychiatry 2014; 48: 722–734. 24744400 10.1177/0004867414531078

[bib18] Fung SJ, Joshi D, Fillman SG, Weickert CS. High white matter neuron density with elevated cortical cytokine expression in schizophrenia. Biol Psychiatry 2014; 75: e5–e7. 23830667 10.1016/j.biopsych.2013.05.031

[bib19] Van Haren NE, Schnack HG, Cahn W, Van den Heuvel MP, Lepage C, Collins L et al. Changes in cortical thickness during the course of illness in schizophrenia. Arch Gen Psychiatry 2011; 68: 871–880. 21893656 10.1001/archgenpsychiatry.2011.88

[bib20] Navari S, Dazzan P. Do antipsychotic drugs affect brain structure? A systematic and critical review of MRI findings. Psychol Med 2009; 39: 1763–1777. 19338710 10.1017/S0033291709005315

[bib21] Vita A, De Peri L, Deste G, Barlati S, Sacchetti E. The effect of antipsychotic treatment on cortical gray matter changes in schizophrenia: does the class matter? A meta-analysis and meta-regression of longitudinal magnetic resonance imaging studies. Biol Psychiatry 2015; 78: 403–412. 25802081 10.1016/j.biopsych.2015.02.008

[bib22] Pillai A, Parikh V, Terry AVJ, Mahadik SP. Long-term antipsychotic treatments and crossover studies in rats: differential effects of typical and atypical agents on the expression of antioxidant enzymes and membrane lipid peroxidation in rat brain. J Psychiatr Res 2007; 41: 372–386. 16564057 10.1016/j.jpsychires.2006.01.011

[bib23] Lipska BK, Khaing ZZ, Weickert CS, Weinberger DR. BDNF mRNA expression in rat hippocampus and prefrontal cortex: effects of neonatal ventral hippocampal damage and antipsychotic drugs. Eur J Neurosci 2001; 14: 135–144. 11488957 10.1046/j.1460-9568.2001.01633.x

[bib24] Sutherland GT, Sheedy D, Stevens J, McCrossin T, Smith CC, van Roijen M et al. The NSW brain tissue resource centre: banking for alcohol and major neuropsychiatric disorders research. Alcohol 2016; 52: 33–39. 27139235 10.1016/j.alcohol.2016.02.005 PMC4855871

[bib25] Davis PJM, Wright EA. A new method for measuring cranial cavity volume and its application to the assessment of cerebral atrophy at autopsy. Neuropath Appl Neurobiol 1977; 3: 341–358.

[bib26] Double KL, Halliday GM, Kril JJ, Harasty JA, Cullen K, Brooks WS et al. Topography of brain atrophy during normal aging and alzheimer's disease. Neurobiol Aging 1996; 17: 513–521. 8832624 10.1016/0197-4580(96)00005-x

[bib27] Sheedy D, Garrick T, Dedova I, Hunt C, Miller R, Sundqvist N et al. An Australian Brain Bank: a critical investment with a high return!. Cell Tissue Bank 2008; 9: 205–216. 18543078 10.1007/s10561-008-9076-1 PMC3391553

[bib28] Hardy JA, Wester P, Winblad B, Gezelius C, Bring G, Eriksson A. The patients dying after long terminal phase have acidotic brains; implications for biochemical measurements on autopsy tissue. J Neural Transm 1985; 61: 253–264. 3989524 10.1007/BF01251916

[bib29] Halliday GM, Double KL, Macdonald V, Kril JJ. Identifying severely atrophic cortical subregions in Alzheimer's disease. Neurobiol Aging 2003; 24: 797–806. 12927762 10.1016/s0197-4580(02)00227-0

[bib30] Kril JJ, Halliday GM, Svoboda MD, Cartwright H. The cerebral cortex is damaged in chronic alcoholics. Neuroscience 1997; 79: 983–998. 9219961 10.1016/s0306-4522(97)00083-3

[bib31] Piguet O, Double KL, Kril JJ, Harasty J, Macdonald V, McRitchie DA et al. White matter loss in healthy ageing: A postmortem analysis. Neurobiol Aging 2009; 30: 1288–1295. 18077060 10.1016/j.neurobiolaging.2007.10.015

[bib32] Gundersen HJ, Jensen EB. The efficiency of systematic sampling in stereology and its prediction. J Microsc 1987; 147: 229–263. 3430576 10.1111/j.1365-2818.1987.tb02837.x

[bib33] Joshi D, Fung SJ, Rothwell A, Weickert CS. Higher gamma-aminobutyric acid neuron density in the white matter of orbital frontal cortex in schizophrenia. Biol Psychiatry 2012; 72: 725–733. 22841514 10.1016/j.biopsych.2012.06.021

[bib34] Weickert CS, Sheedy D, Rothmond DA, Dedova I, Fung SJ, Garrick T et al. Selection of reference gene expression in a schizophrenia brain cohort. Aust N Z J Psychiatry 2010; 44: 59–70. 20073568 10.3109/00048670903393662 PMC2950262

[bib35] Joshi D, Catts VS, Olaya JC, Weickert CS. Relationship between somatostatin and death receptor expression in the orbital frontal cortex in schizophrenia: a postmortem brain mRNA study. Npj Schizophrenia 2015; 1: 14004. 27336026 10.1038/npjschz.2014.4 PMC4849439

[bib36] O'Brien LM, Ziegler DA, Deutsch CK, Frazier JA, Herbert MR, Locascio JJ. Statistical adjustments for brain size in volumetric neuroimaging studies: some practical implications in methods. Psychiatry Res 2011; 193: 113–122. 21684724 10.1016/j.pscychresns.2011.01.007 PMC3510982

[bib37] Courchesne E, Chisum HJ, Townsend J, Cowles A, Covington J, Egaas B et al. Normal brain development and aging: quantitative analysis at *in vivo* MR imaging in healthy volunteers. Radiology 2000; 216: 672–682. 10966694 10.1148/radiology.216.3.r00au37672

[bib38] Semendeferi K, Armstrong E, Schleicher A, Zilles K, Van Hoesen GW. Prefrontal cortex in humans and apes: a comparative study of area 10. Am J Phys Anthropol 2001; 114: 224–241. 11241188 10.1002/1096-8644(200103)114:3<224::AID-AJPA1022>3.0.CO;2-I

[bib39] Bartona RA, Venditti C. Human frontal lobes are not relatively large. Proc Natl Acad Sci USA 2013; 110: 9001–9006. 23671074 10.1073/pnas.1215723110 PMC3670331

[bib40] Cahn W, Hulshoff Pol HE, Lems EB, Van Haren NE, Schnack HG, Van der Linden JA et al. Brain volume changes in first-episode schizophrenia: a 1-year follow-up study. Arch Gen Psychiatry 2002; 59: 1002–1010. 12418933 10.1001/archpsyc.59.11.1002

[bib41] Zipursky RB, Lambe EK, Kapur S, Mikulis DJ. Cerebral gray matter volume deficits in first episode psychosis. Arch Gen Psychiatry 1998; 55: 540–546. 9633673 10.1001/archpsyc.55.6.540

[bib42] Ohtani T, Levitt JJ, Nestor PG, Kawashima T, Asami T, Shenton ME et al. Prefrontal cortex volume deficit in schizophrenia: a new look using 3T MRI with manual parcellation. Schizophr Res 2014; 152: 184–190. 24280350 10.1016/j.schres.2013.10.026

[bib43] Suzuki M, Zhou SY, Takahashi T, Hagino H, Kawasaki Y, Niu L et al. Differential contributions of prefrontal and temporolimbic pathology to mechanisms of psychosis. Brain 2005; 128: 2109–2122. 15930048 10.1093/brain/awh554

[bib44] Schobitz B, de Kloet ER, Sutanto W, Holsboer F. Cellular localization of interleukin 6 mRNA and interleukin 6 receptor mRNA in rat brain. Eur J Neurosci 1993; 5: 1426–1435. 8287192 10.1111/j.1460-9568.1993.tb00210.x

[bib45] Cannella B, Raine CS. Multiple sclerosis: cytokine receptors on oligodendrocytes predict innate regulation. Ann Neurol 2004; 55: 46–57. 14705111 10.1002/ana.10764

[bib46] Giulian D, Baker TJ, Shih LC, Lachman LB. Interleukin 1 of the central nervous system is produced by ameboid microglia. J Exp Med 1986; 164: 594–604. 3487617 10.1084/jem.164.2.594 PMC2188228

[bib47] Knerlich F, Schilling L, Gorlach C, Wahl M, Ehrenreich H, Siren AL. Temporal profile of expression and cellular localization of inducible nitric oxide synthase, interleukin-1beta and interleukin converting enzyme after cryogenic lesion of the rat parietal cortex. Brain Res Mol Brain Res 1999; 68: 73–87. 10320785 10.1016/s0169-328x(99)00064-9

[bib48] Wong M, Licinio J. Localization of interleukin 1 type I receptor mRNA in rat brain. Neuroimmunomodulation 1994; 1: 110–115. 7489320 10.1159/000097143

[bib49] Blasi F, Riccio M, Brogi A, Strazza M, Taddei ML, Romagnoli S et al. Constitutive expression of interleukin-1beta (IL-1beta) in rat oligodendrocytes. Biol Chem 1999; 380: 259–264. 10195433 10.1515/BC.1999.034

[bib50] Erta M, Quintana A, Hidalgo J. Interleukin-6, a major cytokine in the central nervous system. Int J Biol Sci 2012; 8: 1254–1266. 23136554 10.7150/ijbs.4679 PMC3491449

[bib51] Hewett SJ, Jackman NA, Claycomb RJ. Interleukin-1β in central nervous system injury and repair. Eur J Neurodegener Dis 2012; 1: 195–211. 26082912 PMC4465544

[bib52] Baker C, Belbin O, Kalsheker N, Morgan K. SERPINA3 (aka alpha-1-antichymotrypsin). Front Biosci 2007; 12: 2821–2835. 17485262 10.2741/2275

[bib53] Abraham CR. Reactive astrocytes and alpha1-antichymotrypsin in Alzheimer's disease. Neurobiol Aging 2001; 22: 931–936. 11755001 10.1016/s0197-4580(01)00302-5

[bib54] Nilsson LN, Das S, Potter H. Effect of cytokines, dexamethasone and the A/T-signal peptide polymorphism on the expression of alpha(1)-antichymotrypsin in astrocytes: significance for Alzheimer's disease. Neurochem Int 2001; 39: 361–370. 11578771 10.1016/s0197-0186(01)00043-2

[bib55] Kanemaru K, Meckelein B, Marshall DC, Sipe JD, Abraham CR. Synthesis and secretion of active alpha 1-antichymotrypsin by murine primary astrocytes. Neurobiol Aging 1996; 17: 767–771. 8892350 10.1016/0197-4580(96)00111-x

[bib56] Koo EH, Abraham CR, Potter H, Cork LC, Price DL. Developmental expression of alpha 1-antichymotrypsin in brain may be related to astrogliosis. Neurobiol Aging 1991; 12: 495–501. 1837594 10.1016/0197-4580(91)90079-y

[bib57] Ishiguro K, Shoji M, Yamaguchi H, Matsubara E, Ikeda M, Kawarabayashi T et al. Differential expression of alpha 1-antichymotrypsin in the aged human brain. Virchows Arch B Cell Pathol Mol Pathol 1993; 64: 221–227. 10.1007/BF02915116 8287118

[bib58] Arion D, Unger T, Lewis DA, Levitt P, Mirnics K. Molecular evidence for increased expression of genes related to immune and chaperone function in the prefrontal cortex in schizophrenia. Biol Psychiatry 2007; 62: 711–721. 17568569 10.1016/j.biopsych.2006.12.021 PMC2080683

[bib59] Saetre P, Emilsson L, Axelsson E, Kreuger J, Lindholm E, Jazin E. Inflammation-related genes up-regulated in schizophrenia brains. BMC Psychiatry 2007; 7: 1–10. 17822540 10.1186/1471-244X-7-46 PMC2080573

[bib60] Ehrlich LC, Hu S, Sheng WS, Sutton RL, Rockswold GL, Peterson PK et al. Cytokine regulation of human microglial cell IL-8 production. J Immunol 1998; 160: 1944–1948. 9469457

[bib61] Bartha AI, Foster-Barber A, Miller SP, Vigneron DB, Glidden DV, Barkovich AJ et al. Neonatal encephalopathy: association of cytokines with MR spectroscopy and outcome. Pediatr Res 2004; 56: 960–966. 15496611 10.1203/01.PDR.0000144819.45689.BB

[bib62] Dammann O, O'Shea TM. Cytokines and perinatal brain damage. Clin Perinatol 2008; 35: 643–663. 19026332 10.1016/j.clp.2008.07.011 PMC3657129

[bib63] Bloomfield PS, Selvaraj S, Veronese M, Rizzo G, Bertoldo A, Owen DR et al. Microglial activity in people at ultra high risk of psychosis and in schizophrenia; an [(11)C]PBR28 PET brain imaging study. Am J Psychiatry 2016; 173: 44–52. 26472628 10.1176/appi.ajp.2015.14101358 PMC4821370

[bib64] Doorduin J, de Vries EF, Willemsen AT, de Groot JC, Dierckx RA, Klein HC. Neuroinflammation in schizophrenia-related psychosis: a PET study. J Nucl Med 2009; 50: 1801–1807. 19837763 10.2967/jnumed.109.066647

[bib65] Kenk M, Selvanathan T, Rao N, Suridjan I, Rusjan P, Remington G et al. Imaging neuroinflammation in gray and white matter in schizophrenia: an in-vivo PET study with [18F]-FEPPA. Schizophr Bull 2015; 41: 85–93. 25385788 10.1093/schbul/sbu157 PMC4266311

[bib66] Thompson M, Weickert CS, Wyatt E, Webster MJ. Decreased glutamic acid decarboxylase67 mRNA expression in multiple brain areas of patients with schizophrenia and mood disorders. J Psychiatr Res 2009; 43: 970–977. 19321177 10.1016/j.jpsychires.2009.02.005

[bib67] Al-Amin MM, Nasir Uddin MM, Mahmud Reza H. Effects of antipsychotics on the inflammatory response system of patients with schizophrenia in peripheral blood mononuclear cell cultures. Clin Psychopharmacol Neurosci 2013; 11: 144–151. 24465251 10.9758/cpn.2013.11.3.144 PMC3897763

[bib68] Maes M, Bocchio Chiavetto L, Bignotti S, Battisa Tura G, Pioli R, Boin F et al. Effects of atypical antipsychotics on the inflammatory response system in schizophrenic patients resistant to treatment with typical neuroleptics. Eur Neuropsychopharmacol 2000; 10: 119–124. 10706993 10.1016/s0924-977x(99)00062-0

[bib69] Klein TW. Cannabinoid-based drugs as anti-inflammatory therapeutics. Nat Rev Immunol 2005; 5: 400–411. 15864274 10.1038/nri1602

[bib70] Karama S, Ducharme S, Corley J, Chouinard-Decorte F, Starr JM, Wardlaw JM et al. Cigarette smoking and thinning of the brain's cortex. Mol Psychiatry 2015; 20: 778–785. 25666755 10.1038/mp.2014.187 PMC4430302

[bib71] Schiffer B, Müller BW, Scherbaum N, Michael F, Wiltfang J, Leygrat N et al. Impulsivity-related brain volume deficits in schizophrenia-addiction comorbidity. Brain 2010; 133: 3093–3103. 20647266 10.1093/brain/awq153

[bib72] Winklbaur B, Ebner N, Sachs G, Thau K, Fischer G. Substance abuse in patients with schizophrenia. Dialogues Clin Neurosci 2006; 8: 37–43. 16640112 10.31887/DCNS.2006.8.1/bwinklbaur PMC3181760

[bib73] Heckers S, Konradi C. Hippocampal neurons in schizophrenia. J Neural Transm 2002; 109: 891–905. 12111476 10.1007/s007020200073 PMC4205576

