# Supplementary material for: Cortical grey matter volume reduction in people with schizophrenia is associated with neuro-inflammation
Source: Transl Psychiatry. 2016 Dec 13;6(12):e982–. doi: 10.1038/tp.2016.238 (PMC5290336; doi:10.1038/tp.2016.238)
Supplement: Supplementary Information [file tp2016238x1.docx]

**Supplementary Figure 1.** Graph of superior frontal gyrus (SFG) data points with 95% boundary lines. Data points outside the 95% boundary line were visually identified, sections from outlier cases were reviewed by two investigators (VC & YZ) to confirm regional boundaries and the volume was re-measured. If the data points remained outside of the 95% boundary line following the recount, this case was excluded from the analysis of that particular region.

**A.** Schizophrenia. **B.** Unaffected controls. Following review, cases 228, 230, and 775 were situated outside the 95% confidence interval boundary line, and were therefore not included in the SFG data analysis.


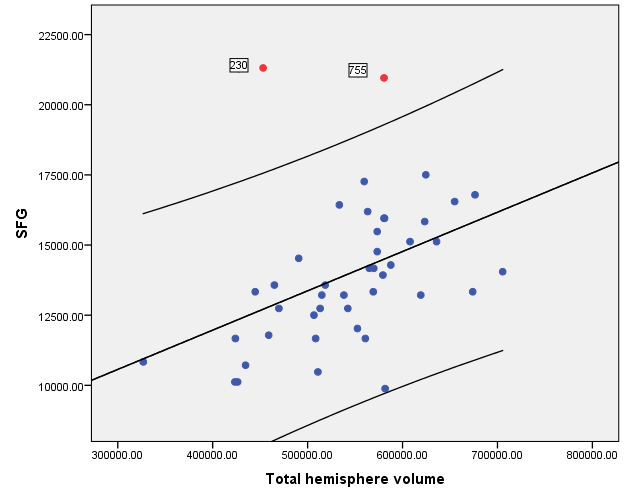


**A**


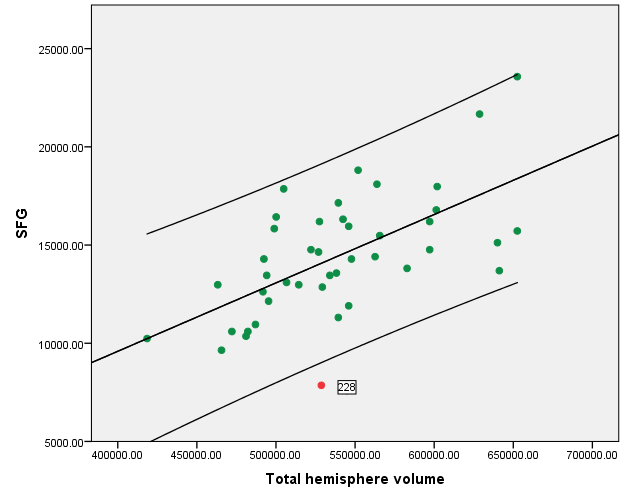


**B**

**Supplementary Table 1.** Description of the subgroups for whom both volume and inflammation data (DLPFC) were available.

|  | Controls | | | Schizophrenia | | | Statistics | | | |
| --- | --- | --- | --- | --- | --- | --- | --- | --- | --- | --- |
|  | N | mean | SD | N | mean | SD | *X^2^* | *t* | *df* | *p* |
| Male | 18 | --- | --- | 18 | --- | --- | 0.696 | --- | 1 | 0.549 |
| Female | 6 | --- | --- | 10 | --- | --- |  |  |  |  |
| Left Hemisphere | 15 | --- | --- | 13 | --- | --- | 1.343 | --- | 1 | 0.278 |
| Right Hemisphere | 9 | --- | --- | 15 | --- | --- |  |  |  |  |
| Age (years) | --- | 52.8 | 13.1 | --- | 53.0 | 13.6 | --- | 0.035 | 50 | 0.972 |
| PMI | --- | 25.9 | 12.1 | --- | 29.3 | 14.1 | --- | 0.936 | 50 | 0.354 |
| Brain weight  (grams) | --- | 1403.6 | 113.7 | --- | 1398.0 | 143.3 | --- | -0.115 | 50 | 0.877 |
| Tissue pH | --- | 6.5 | 0.3 | --- | 6.5 | 0.2 | --- | -0.299 | 50 | 0.766 |
| RIN | --- | 7.28 | 0.65 | --- | 7.23 | 0.57 | --- | 0.323 | 50 | 0.748 |
| DLPFC: High Inflammation | 2 | --- | --- | 10 | --- | --- | 5.458 | --- | 1 | 0.024 |
| DLPFC: Low Inflammation | 22 | --- | --- | 18 | --- | --- |  |  |  |  |

SD: standard deviation; PMI: postmortem interval; RIN: RNA integrity number.

* Unknown cases were not involved in statistical analysis;

**Supplementary Table 2.** Pearson correlation (r): Brain regional volume versus age, total hemisphere volume, brain weight, PMI, and tissue pH.

|  | Age | | Total hemisphere volume | | Brain weight | | PMI | | Tissue pH | | Displacement volume | |
| --- | --- | --- | --- | --- | --- | --- | --- | --- | --- | --- | --- | --- |
|  | r | *p* | r | *p* | r | *p* | r | *p* | r | *p* | r | *p* |
| Brain weight | -0.391 | <0.001 | 0.675 | <0.001 | --- | --- | 0.070 | 0.505 | -0.024 | 0.823 | 0.978 | <0.001 |
| Displacement volume | -0.355 | 0.001 | 0.723 | <0.001 | 0.978 | <0.001 | 0.074 | 0.492 | 0.220 | 0.039 | --- | --- |
| Total hemisphere volume | -0.353 | 0.001 | --- | --- | 0.675 | <0.001 | 0.105 | 0.320 | -0.107 | 0.311 | 0.723 | <0.001 |
| White matter | -0.389 | <0.001 | 0.929 | <0.001 | 0.609 | <0.001 | 0.018 | 0.870 | -0.115 | 0.285 | 0.660 | <0.001 |
| Cortical Grey matter | -0.280 | 0.008 | 0.956 | <0.001 | 0.661 | <0.001 | 0.144 | 0.177 | -0.078 | 0.475 | 0.706 | <0.001 |
| PFC | -0.365 | 0.001 | 0.818 | <0.001 | 0.603 | <0.001 | 0.105 | 0.347 | 0.013 | 0.911 | 0.647 | <0.001 |
| Frontal pole | -0.244 | 0.021 | 0.503 | <0.001 | 0.374 | <0.001 | 0.048 | 0.658 | 0.004 | 0.970 | 0.376 | <0.001 |
| SFG | -0.258 | 0.018 | 0.576 | <0.001 | 0.486 | <0.001 | 0.039 | 0.726 | -0.005 | 0.963 | 0.527 | <0.001 |
| MFG | -0.295 | 0.005 | 0.583 | <0.001 | 0.483 | <0.001 | 0.167 | 0.121 | 0.051 | 0.637 | 0.521 | <0.001 |
| IFG | -0.258 | 0.015 | 0.531 | <0.001 | 0.479 | <0.001 | 0.082 | 0.447 | 0.043 | 0.689 | 0.517 | <0.001 |
| OFC | -0.214 | 0.044 | 0.620 | <0.001 | 0.496 | <0.001 | 0.113 | 0.293 | -0.191 | 0.074 | 0.522 | <0.001 |

PMI: postmortem interval; PFC: prefrontal cortex; SFG: superior frontal gyrus; MFG: middle frontal gyrus; IFG: inferior frontal gyrus; OFC: orbitofrontal cortex

**Supplementary Table 3.** Effect of gender, hemisphere, and diagnosis on brain weight and volume.

|  | **Gender** | | | | | | |  | **Hemisphere** | | | | | | |  | **Diagnosis** | | | | | | |
| --- | --- | --- | --- | --- | --- | --- | --- | --- | --- | --- | --- | --- | --- | --- | --- | --- | --- | --- | --- | --- | --- | --- | --- |
|  | Male | | Female | | Analysis | | |  | Left | | Right | | Analysis | | |  | Controls | | Schizophrenia | | Analysis | | |
|  |  |  |  |  |  |  |  |  |  |  |  |  |  |  |  |  |  |  |  |  | F | df | p |
|  | mean | SD | mean | SD | F | df | p |  | mean | SD | mean | SD | F | df | p |  | mean | SD | mean | SD |  |  |  |
| Brain Weight (g)* | 1487 | 107 | 1284 | 122 | 48.703 | 1, 89 | <0.001 |  | --- | --- | --- | --- | --- | --- | --- |  | 1431 | 143 | 1411 | 152 | 0.689 | 1, 89 | 0.409 |
| Displa-cement (cm^3^)* | 1471 | 108 | 1288 | 130 | 36.188 | 1, 86 | <0.001 |  | --- | --- | --- | --- | --- | --- | --- |  | 1415 | 140 | 1403 | 149 | 0.361 | 1, 86 | 0.550 |
| Total Hemisphere (cm^3^)* | 560 | 56 | 504 | 70 | 9.540 | 1, 89 | 0.003 |  | 545 | 69 | 539 | 63 | 0.233 | 1, 89 | 0.631 |  | 540 | 54 | 544 | 76 | 0.061 | 1, 89 | 0.805 |
| Grey Matter  (cm^3^)# | 312 | 32 | 284 | 41 | 7.494 | 1, 86 | 0.008 |  | 305 | 38 | 300 | 37 | 0.738 | 1, 85 | 0.393 |  | 305 | 30 | 301 | 42 | 4.478 | 1, 85 | 0.037 |
| White Matter (cm^3^)# | 220 | 26 | 194 | 33 | 8.394 | 1, 86 | 0.005 |  | 212 | 32 | 211 | 29 | 0.428 | 1, 85 | 0.515 |  | 209 | 25 | 214 | 35 | 3.364 | 1, 85 | 0.070 |
| PFC  (cm^3^)# | 77 | 13 | 70 | 11 | 2.028 | 1, 80 | 0.158 |  | 76 | 13 | 73 | 12 | 1.457 | 1, 80 | 0.231 |  | 74 | 11 | 76 | 14 | 0.031 | 1, 79 | 0.861 |
| Frontal pole  (cm^3^)# | 18 | 5 | 16 | 6 | 0.436 | 1, 86 | 0.511 |  | 17 | 6 | 17 | 5 | 0.040 | 1, 85 | 0.841 |  | 17 | 5 | 18 | 7 | 0.565 | 1, 85 | 0.454 |
| SFG  (cm^3^)# | 14 | 3 | 13 | 2 | 0.842 | 1, 81 | 0.362 |  | 15 | 3 | 14 | 2 | 3.604 | 1, 80 | 0.061 |  | 15 | 3 | 14 | 2 | 5.700 | 1, 80 | 0.019 |
| MFG (cm^3^)# | 16 | 3 | 14 | 2 | 2.126 | 1, 85 | 0.149 |  | 16 | 3 | 15 | 3 | 3.743 | 1, 84 | 0.056 |  | 15 | 3 | 15 | 3 | 0.033 | 1, 84 | 0.856 |
| IFG  (cm^3^)# | 15 | 2 | 14 | 2 | 1.816 | 1, 85 | 0.181 |  | 15 | 3 | 15 | 2 | 1.542 | 1, 84 | 0.218 |  | 15 | 2 | 15 | 3 | 0.774 | 1, 84 | 0.382 |
| OFC  (cm^3^)# | 13 | 2 | 12 | 2 | 3.900 | 1, 86 | 0.051 |  | 13 | 2 | 12 | 2 | .0606 | 1, 85 | 0.438 |  | 13 | 2 | 12 | 2 | 0.723 | 1, 85 | 0.398 |

*Brain weight, displacement and total hemisphere volume were co-varied with age.

Grey matter, white matter, PFC, frontal pole, SFG, MFG, IFG, and OFC volume were co-varied with age and total hemisphere volume.

SD: standard deviation; PFC: prefrontal cortex; SFG: superior frontal gyrus; MFG: middle frontal gyrus; IFG: inferior frontal gyrus; OFC: orbitofrontal cortex.

**Supplementary Table 4.** **Pearson’s correlation (r):** Cortical grey matter and superior frontal gyrus (SFG) volumes versus cytokines (Interleukin-6, SERPIN3A, Interleukin-1β, Interleukin-8) mRNA expression in dorsolateral prefrontal cortex. **Spearman’s correlation (rho):** Cortical grey matter and SFG volumes versus antipsychotic medication doses (chlorpromazine equivalents). (*p<0.05, **p<0.01).

|  | | Grey Matter | | SFG | |
| --- | --- | --- | --- | --- | --- |
|  |  | All | Schizophrenia | All | Schizophrenia |
| Interleukin 6 | *r* | -0.361* | -0.506** | -0.512** | -0.700** |
| SERPIN3A | *r* | -0.395* | -0.637** | -0.341* | -0.652** |
| Interleukin 1β | *r* | -0.051 | -0.323 | -0.092 | -0.563** |
| Interleukin 8 | *r* | 0.103 | -0.125 | 0.149 | 0.113 |
| Duration of illness | *r* | --- | -0.428* | --- | -0.438* |
| Chlorpromazine  life time | *rho* | --- | -0.424* | --- | -0.479** |
| Chlorpromazine  daily mean | *rho* | --- | -0.362* | --- | -0.404* |

**Supplementary Figure 2.** Correlations between brain volumes and dorsolateral prefrontal cortex (DLPFC) inflammatory markers. Correlation of cortical grey matter volume with (**A**) interleukin (IL)-6 and (**B**) SERPINA3; correlation of superior frontal gyrus volume with (**C**) interleukin (IL)-6 and (**D**) SERPINA3. Blue dots indicate controls and red dots are individuals with schizophrenia. Data of inflammatory markers are log10 transformed data.

| **A** | 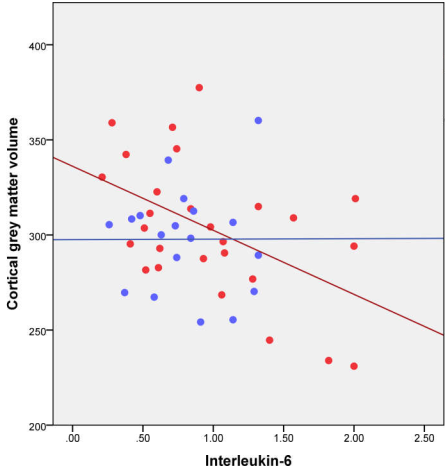 |
| --- | --- |
| **B** | 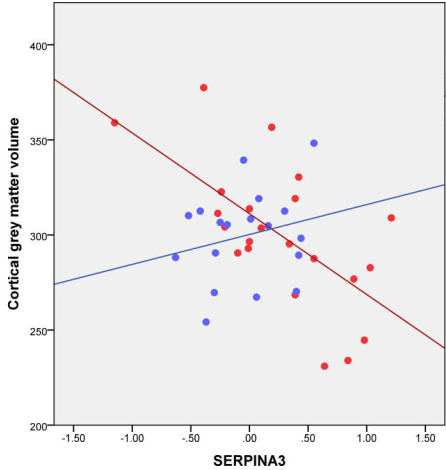 |
| **C** |  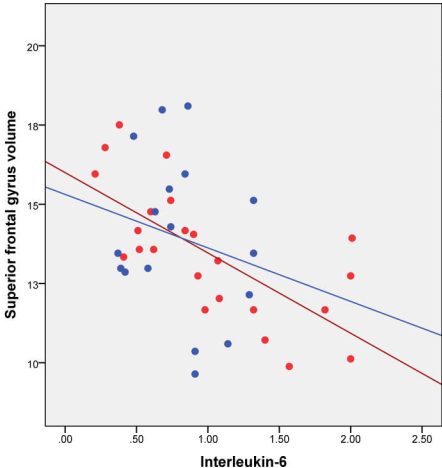  |
| **D** | 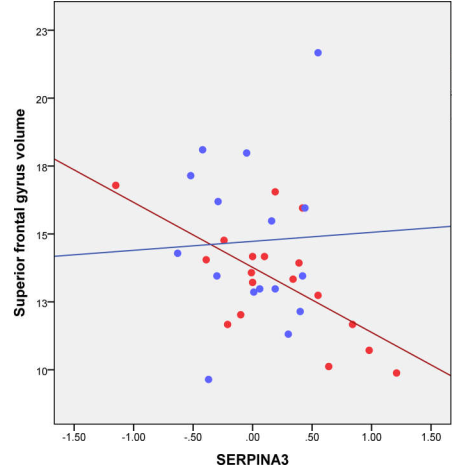 |

**Supplementary Table 5.** Correlations of OFC inflammatory markers with demographics. As some demographic data are abnormal distribution, Spearman’s correlation (rho) was used for analysis.

|  | SERPIN3A | | Interleukin 6 | | Interleukin 1β | | Interleukin 8 | |
| --- | --- | --- | --- | --- | --- | --- | --- | --- |
|  | rho | *p* | rho | *p* | rho | *p* | rho | *p* |
| Age | -0.458 | 0.001 | -0.247 | 0.032 | 0.143 | 0.220 | -0.205 | 0.076 |
| PMI | -0.141 | 0.226 | -0.166 | 0.156 | -0.054 | 0.643 | 0.089 | 0.445 |
| pH | -0.270 | 0.025 | -0.553 | 0.001 | -0.072 | 0.554 | -0.205 | 0.089 |
| RIN | -0.171 | 0.142 | -0.264 | 0.022 | 0.047 | 0.687 | -0.119 | 0.306 |
| Antipsychotics  Life time* | 0.397 | 0.016 | 0.464 | 0.004 | 0.008 | 0.962 | -0.136 | 0.428 |
| Antipsychotics  daily mean* | 0.311 | 0.065 | 0.399 | 0.016 | 0.145 | 0.400 | -0.024 | 0.892 |

* Only in schizophrenia group.

PMI: postmortem interval; RIN: RNA integrity number.

**Supplementary Figure 3.** The distribution of levels inflammatory markers in high and low groups of the OFC cohort. All expression data were normalized to housekeeping genes.
(**A**) SERPINA3 mRNA; (**B**) Interleukin-1β mRNA; (**C**) Interleukin-6 mRNA;
(**D**) Interleukin-8 mRNA. Y-axis is log scale.

| **A** |  | **B** |  |
| --- | --- | --- | --- |
| **C** |  | **D** |  |
